# Changes in Urologic Operative Practice at the Beginning of the COVID-19 Pandemic in a Large, National Cohort

**DOI:** 10.3389/fonc.2021.684787

**Published:** 2021-05-07

**Authors:** Patrick Lewicki, Camilo Arenas-Gallo, Spyridon P. Basourakos, Nahid Punjani, Siv Venkat, Douglas S. Scherr, Jim C. Hu, Jonathan E. Shoag

**Affiliations:** ^1^ Department of Urology, New York Presbyterian Hospital, Weill Cornell Medicine, New York, NY, United States; ^2^ Department of Urology, University Hospitals Cleveland Medical Center, Case Western Reserve University School of Medicine, Cleveland, OH, United States

**Keywords:** (MeSH): COVID-19, surgical procedures, urologic surgical procedures, urology, oncologic urology

## Abstract

**Objective:**

To analyze population-level changes in operative practice since the onset of the COVID-19 pandemic to contextualize observations made by individual practices and optimize future responses.

**Materials and Methods:**

This US retrospective analysis used the Premier Perspectives Database. We investigated changes in operative volume through March 2020. Baseline operative volume for urologic surgery was calculated using data from the preceding 12 months and compared on a total and by procedure basis. Multivariable linear regression was used to identify hospital-level predictors of change in response to the pandemic.

**Results:**

At baseline, we captured 23,788 urologic procedural encounters per month as compared with 19,071 during March 2020– a 19.9% decrease. Urologic oncology-related cases were relatively preserved as compared to others (average change in March 2020: +1.1% versus -32.2%). Northeastern (β = -5.66, 95% confidence interval [CI]: -10.2 to -1.18, p = 0.013) and Midwestern hospitals (β = -4.17, 95% CI: -7.89 to -0.45, p = 0.027; both with South as reference region), and those with an increasing percentage of patients insured by Medicaid (β= -0.17 per percentage point, 95% CI: -0.33 to -0.01, p = 0.04) experienced a significantly larger decrease in volume.

**Conclusions:**

There was a 20% decline in urologic operative volume in March 2020, compared with baseline, that preferentially affected hospitals serving Medicaid patients, and those in Northeast and Midwest. In the face of varying mandates on elective surgery, widespread declines in operative volume may also represent hesitancy on behalf of patients to interface with healthcare during the pandemic.

## Introduction

The COVID-19 pandemic and consequent re-allocation of medical resources has impacted the practice of urology worldwide. At the beginning of the outbreak, elective surgery in many locales was paused, and patients were reluctant to present to care, even when medically necessary (1). Providers across a range of non-COVID related specialties were forced to prioritize certain patients and conditions over others, which is reflected in guidelines from the urologic literature proposing triage algorithms for patient management (2–4).

In spite of these recommendations, the extent to which they were adhered to by the urologic community at large is sparsely described, particularly across diverse hospitals (e.g., varying geography, academic affiliation, and patient population). Complicating this speculation is the heterogeneous toll of the pandemic between and within countries, varying local and state governmental mandates on elective surgery, and inconsistent definitions within institutions of what constitutes an emergent versus urgent or elective case (5). Triage patterns for lower-acuity oncologic diagnoses such as prostate cancer or small renal tumors are fraught with uncertainty given the indeterminate but likely low risks of postponing surgery.

While the subject of frequent speculation, the actual impact of the COVID-19 pandemic on urologic operative practice is unknown. Understanding the consequences of the pandemic will teach invaluable lessons for future preparedness and provide useful context for individual practices attempting to understand changes in their own operative volume. Here, we used data from a large United States (US)-based cohort to assess changes in operative volume during March 2020, corresponding to the onset of the COVID-19 pandemic, relative to baseline.

## Materials and Methods

### Study Cohort

Premier Healthcare Database (Premier) is an all-payer database that captures outcomes from approximately 20% of hospital admissions (6). Data captured includes patient encounters through March 2020 and therefore provides an opportunity to investigate changes in urologic care at the beginning of the pandemic within the US.

The study cohort included patients undergoing surgery in either an ambulatory surgery or inpatient setting under the care of a urologist. Variables specifying patient setting and information on provider specialty are both available in Premier and were used to define the overall cohort, without selecting for specific procedures. To understand changes in specific procedures, Current Procedural Terminology (CPT) and International Classification of Diseases-10 Procedure Coding System (ICD-10-PCS) codes (Supplementary list) were used to identify surgeries of interest within the overall cohort. In-office procedures were not studied as they are incompletely capture in Premier relative to in-hospital procedures.

Our analysis included data from the preceding calendar year to account for seasonal variation and trends in patient volume, and therefore the study period captures patient discharges from March 2019 to March 2020. Additionally, given changing hospital membership in Premier over time, we included encounters from only those centers submitting data throughout the entire study period.

### Outcomes and Exposures

Our primary outcome of interest was change in operative volume in March 2020 relative to baseline. Total operative volume, and volume by procedure and procedure-based groupings were investigated.

Baseline operative volume was calculated using encounters from March 2019 to February 2020 and compared with March 2020, representing “pre-COVID” and “early COVID” periods, respectively, within the US. Current Procedural Terminology (CPT) and International Classification of Diseases-10 Procedure Coding System (ICD-10-PCS) codes were used to identify selected procedures (**Supplementary List**).

Surgeries were grouped into the following categories: oncology, voiding dysfunction, endourology/stone disease, and penoscrotal, and separately as elective versus urgent. Urgent cases included surgery for higher risk cancers (radical cystectomy, nephroureterectomy, radical nephrectomy, trans-urethral resection of bladder tumor, and radical orchiectomy). Elective cases included penile prosthesis placement, sling procedures, circumcision, hydrocelectomy, and varicocelectomy.

### Statistical Analysis

Descriptive statistics were tabulated for patient demographics and compared between patients undergoing surgery in March 2020 and March 2019 to February 2020. Continuous and categorical variables were compared with Wilcoxon rank-sum and chi-square test, respectively. Selected procedures were identified and their case counts during March 2020 were compared with baseline and represented as a percentage (of baseline volume).

Multivariable linear regression models were constructed to identify hospital-level predictors of changes in total, urgent, and elective operative volume in March 2020 relative to baseline average. Final models accounted for average operative encounter volume, US Census region, urbanicity, academic affiliation, baseline percent of patients of African-American race and baseline percent of patients with Medicaid; models are included in Supplement. Two-sided p-values of less than 0.05 were considered significant. Analysis was performed in R (version 4.0.1, Foundation for Statistical Computing, Vienna, Austria).

## Results

The final patient cohort included 304,531 patient encounters for urologic surgery at 364 hospitals from March 2019 to March 2020. Baseline average volume was 23,788 procedural encounters per month (65.4 per hospital per month), compared with 19,071 (52.4 per hospital) during March 2020– a 19.9% decrease.

**Table 1** compares patient demographic information between patients undergoing surgery in March 2020 and March 2019-February 2020. No significant difference was seen between time periods with respect to age, gender, and race. A significant difference in insurance type was noted, with fewer Medicaid (and more self pay and “other” insurance) patients undergoing surgery in March 2020. Additionally, significant differences were noted in geographic region (more patients in South, fewer in other regions), urbanicity (fewer patients in Urban settings), and academic status (fewer patients in teaching hospitals).

**Table 1:** Patient characteristics by stratified by study period (March 2019-February 2020, representing baseline, versus March 2020, representing “early COVID-19 pandemic”).

Characteristic	March 2019- February 2020N = 285,460, 94%*^1^*	March 2020N = 19,071, 6.3%*^1^*	p-value*^2^*
**Age**	64 (52, 73)	64 (52, 73)	0.4
**Gender**			0.8
Female	94,643 (94%)	6,354 (6.3%)	
Male	190,812 (94%)	12,717 (6.2%)	
Unknown	5 (100%)	0 (0%)	
**Race**			0.3
Black	22,588 (94%)	1,504 (6.2%)	
White	219,412 (94%)	14,689 (6.3%)	
Hispanic	16,172 (94%)	1,019 (5.9%)	
Other	24,515 (94%)	1,680 (6.4%)	
Unknown	2,773 (94%)	179 (6.1%)	
**Insurance**			**0.007**
Private	96,298 (94%)	6,429 (6.3%)	
Medicare	144,713 (94%)	9,636 (6.2%)	
Medicaid	27,811 (94%)	1,787 (6.0%)	
Self Pay	5,256 (93%)	406 (7.2%)	
Other	11,382 (93%)	813 (6.7%)	
**Region**			**<0.001**
Northeast	52,533 (94%)	3,184 (5.7%)	
Midwest	66,242 (94%)	4,261 (6.0%)	
South	140,126 (93%)	9,904 (6.6%)	
West	26,559 (94%)	1,722 (6.1%)	
**Urbanicity**			**<0.001**
Rural	46,028 (93%)	3,260 (6.6%)	
Urban	239,432 (94%)	15,811 (6.2%)	
**Academic status**			**<0.001**
Academic	139,644 (94%)	8,891 (6.0%)	
Non-academic	145,816 (93%)	10,180 (6.5%)	

^1^Statistics presented: median (IQR); n (%).

^2^Statistical tests performed: Wilcoxon rank-sum test; chi-square test of independence.

Continuous and categorical variables are represented as median (IQR) and number (percent), respectively. Statistics were compared with t-test and chi-square test for continuous and categorical variables as appropriate. Bold p-values are statistically significant.

**Figure 1:**
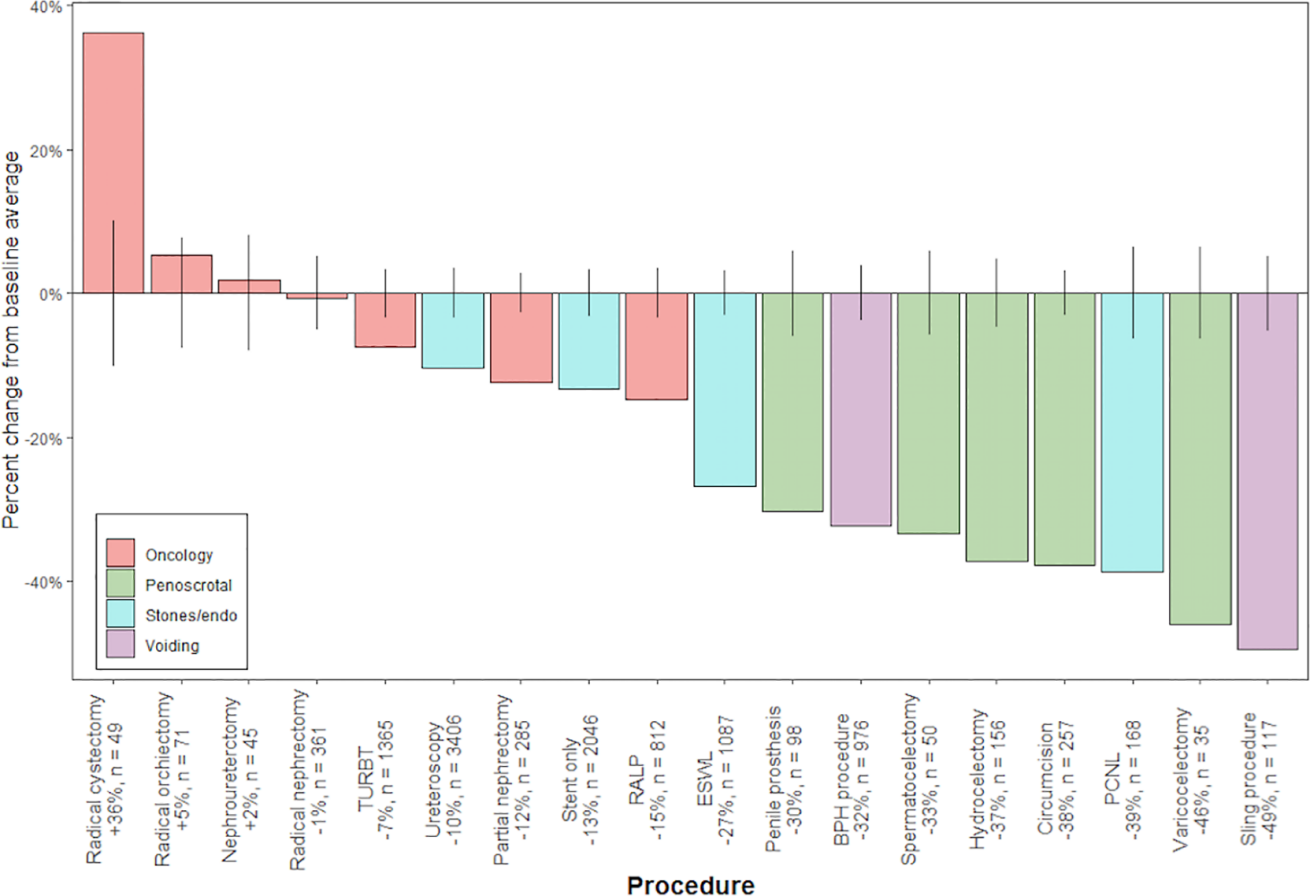
Percent change from average baseline volume during March 2020, by procedure and case type (oncology, penoscrotal surgery, stone disease/endourology, voiding dysfunction). Number of cases during March 2020 is represented by “n”. Error bars correspond to 95% confidence interval for estimate of average baseline volume.

**Figure 1** represents, on a by procedure basis, case volume during March 2020 as a percentage of baseline volume. Positive values represent an increase in case volume during March 2020 relative to baseline, while negative values similarly represent a decrease in case volume. Individual procedures are color-coded by sub-specialty focus. Oncology cases were relatively preserved as compared to other categories (Average change +1.1% versus -32.2%). Amongst oncologic surgeries, partial nephrectomy and robot-assisted laparoscopic prostatectomy saw the largest decreases (-12% and -15%, respectively).

Next, we sought to determine whether hospital demographics independently impacted changes in case volume; in particular, if there was a varying influence of geography, urbanicity, academic status, and patient population on changes in elective cases versus more urgent cases.

On multivariable linear regression, Northeastern (β = -5.66, 95% confidence interval [CI]: -10.2 to -1.18, p = 0.013) and Midwestern region (β = -4.17, 95% CI: -7.89 to -0.45, p = 0.027; both with South as reference region), and increasing percentage of patients with Medicaid (β= -0.17 per percentage point, 95% CI: -0.33 to -0.01, p = 0.04) were significantly correlated with a decrease in total operative volume during March 2020 relative to baseline

Regarding elective cases specifically (as defined above), Northeast and Midwest region alone predicted change in volume (NE: β = -0.53, 95% CI: -1.02 to -0.03, p = 0.037; MW: β = -0.55, 95% CI: -0.94 to -0.15, p = 0.007). Notably, no significant hospital-level predictors of variability in urgent case volume were identified (**Supplementary Table**). Similarly, hospital demographics were not correlated with a change in robotic prostatectomy or partial nephrectomy volume.

**Figure 2:**
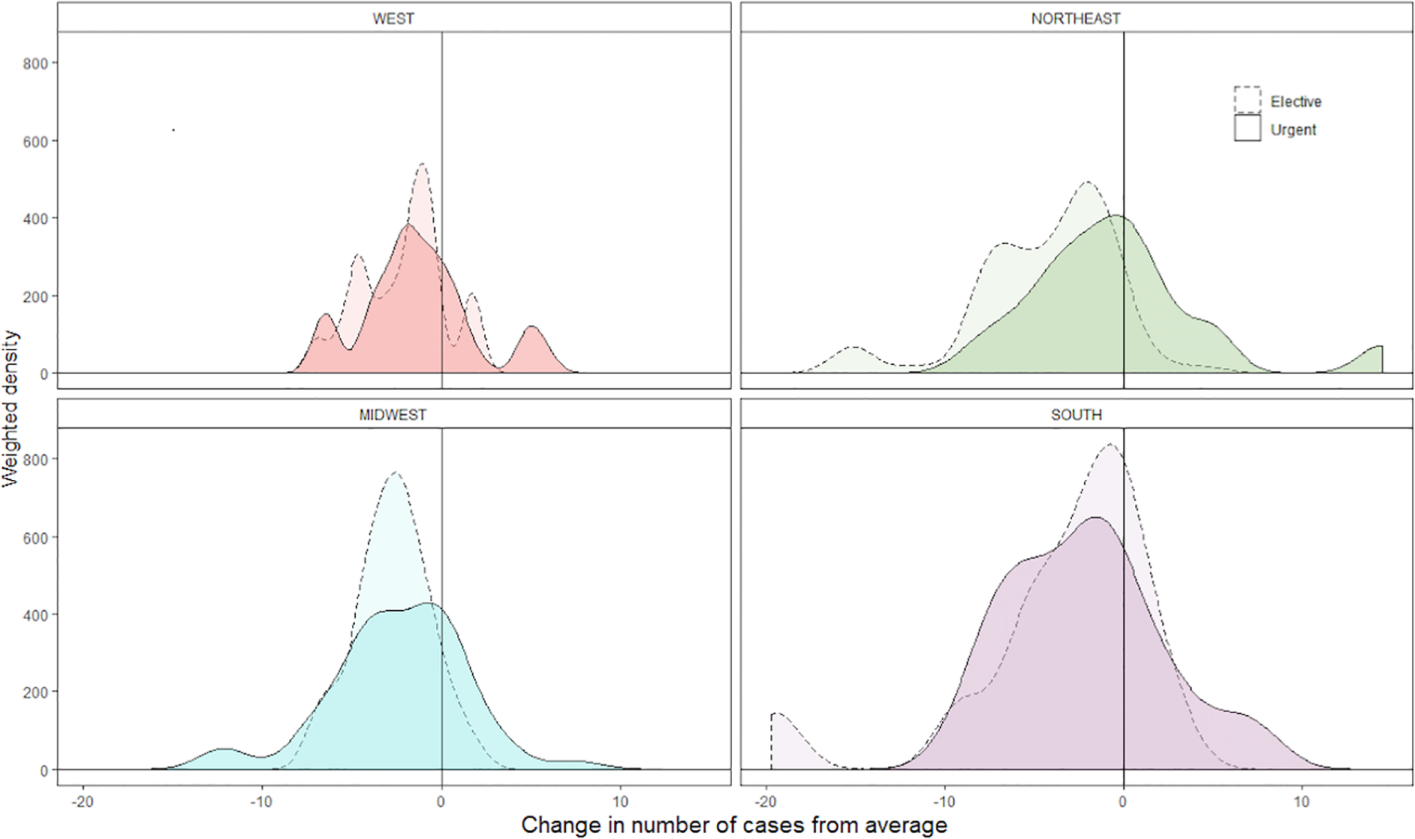
Density plot representing the distribution of per hospital change in elective and urgent operative volume, grouped by US census region. Hospitals in the top decile of baseline operative volume are included. Higher peaks represent a greater concentration of hospitals experiencing a given magnitude of change. Results are weighted by average baseline hospital operative volume. Scale is fixed between all plots.

**Figure 2** shows, for hospitals in the top decile of operative volume, a weighted density plot of the distribution of change in elective and urgent case volume in March 2020 relative to baseline highlighting the wide, variable range of changes in elective case volume throughout the US.

## Discussion

A population-level estimate of the change in operative volume experienced during the COVID-19 pandemic has been difficult to generate. While many providers have observed first-hand a general decrease in urology patient volume, the magnitude of change and the extent to which changes were observed across numerous procedures, diagnoses, and provider demographics has been sparsely described on a national level.

Existing literature largely draws on individual or few-institution group reports. For example, an early from Italy, where the first outbreak of COVID-19 had a very high impact on the hospital network, demonstrated a near complete transition of urological consultation to online or phone interviews (7). The group also described the reduction in prostate cancer treatments like radiotherapy (84.6%) and radical prostatectomy (63.3% reduction) due to a limited availability of anesthesiologists and ventilators (7).

Here, we describe an approximately 20% decrease in operative volume during March 2020, relative to baseline. On a by-procedure basis, oncology cases were relatively preserved compared with all others, in line with published recommendations that higher risk cancer surgery be given priority over other elective cases. Similarly, an appropriate, large decrease in operative volume for elective procedures (e.g., penile prosthesis or sling placement) was observed. This is consistent with previous work from our group that described similar decrease during COVID pandemic in urology-specific emergency room visits (19.4%), admissions to a urology service (19.3%) and ambulatory urology surgeries (22.9%) (8). In this regard, the urology community at large adhered, deliberately or otherwise, to expert guidelines. Interestingly, robotic prostatectomy and partial nephrectomy saw intermediate declines in case volume, which did not vary significantly across geographic region, suggesting that these conditions were managed more similarly to high-acuity oncologic diagnoses during the early phases of the outbreak. Furthermore, large decreases were observed in cases related to voiding dysfunction and andrology, while endourologic procedures decreased slightly less, likely as a consequence of more urgent management of nephrolithiasis.

Geography was a significant predictor of change in overall and elective case volume; percent of patients with Medicaid was correlated with a decrease in overall volume. These results likely reflect the heterogeneous spread of COVID-19 within the US, and the disproportionate effect of the virus across socioeconomic demographics within the same locale. For example, both elective and overall case volume was decreased in the Northeast and Midwest US, two regions with large metropolitan populations where the virus took an early foothold. These decreases were observed relative to the Southern US, where cases did not increase until April and later, with a few exceptions. Additionally, decreased case volume amongst hospitals caring for more Medicaid patients highlights an additional potential mechanism by which the socioeconomically disadvantaged were disproportionately impacted by COVID-19. Finally, there exists substantial variation in the management of elective cases when studied on a by-hospital basis, as seen in **Figure 2**, likely representing variability between institutions in restrictions on non-emergent surgery.

While statistically significant, the magnitude of the correlation coefficients observed here also emphasizes a decrease in total and elective operative volume across all regions and hospital characteristics studied. Given the different governmental and institutional guidelines on elective surgery, the widespread decreases in operative volume likely reflect the reluctance of patients to seek medical care during the pandemic and the preference of outpatient over in-hospital treatments, as described in an analysis of online patient support group discussions of patient concerns and preferences for urolithiasis care during the COVID-19 pandemic (9).

The COVID-19 pandemic also impacted urologic training, which depends on exposure to urologic care. One report from Italy described an up to 81.2% reduction for “clinical” activities, and 62.1% for “surgical” activities amongst residents, especially noticeable in the last years of residency (10). However, some see in this unfortunate pandemic an opportunity to implement innovative solutions in residency programs that can help in the future to close the educational gap and become a regular component of the training of urology residents (11).

Our study is not without limitations and must be interpreted within the context of its design. First, there is a paucity of additional clinical data to adjudicate the necessity of operative intervention versus conservative management. We also lack granularity of procedure date to distinguish early from late March.

Data from April and beyond are lacking, although changes specifically from the onset of the pandemic can inform triage patterns in reaction to natural disasters. As the pandemic continues, it is difficult to know with certainty the most appropriate time for a final analysis of its impact. Interim analyses such as this one provide context for those currently facing outbreaks and planning future studies.

In spite of these limitations, we believe our findings provide insight into the pandemic’s impact on urologic operative practice that has not previously been described. Particularly, a comparison of changes in volume on a by-procedure basis sheds light on adherence to published guidance, as well as the heterogeneous geographic and socioeconomic spread of the pandemic. Long-term follow-up will be necessary to determine COVID-19’s final toll on urology.

## Conclusions

Population-level data describing changes in urologic operative practice during the COVID-19 pandemic are limited. Here, data from a large, national, all-payer database describes an approximately 20% decrease in procedures during March 2020, relative to the preceding year. Urologic oncology cases were relatively preserved compared with others, in line with published guidance. Hospital region and percent of patients with Medicaid were correlated with changes in total volume, highlighting the pandemic’s heterogeneous impact in its early phases. These data contextualize the observations of individual providers and inform triage patters in future disaster response planning.

## Data Availability Statement

The original contributions presented in the study are included in the article/**Supplementary Material**. Further inquiries can be directed to the corresponding author.

## Author Contributions

Study conception and design: PL, SB, JS. Acquisition of data: PL, SB, NP, SV, DS, JH, JS. Analysis and interpretation of data: PL, CA-G, SB, NP, SV, DS, JH, JS. Drafting of manuscript: PL, CA-G, SB, NP, SV, DS, JH, JS. Critical revision: PL, CA-G, SB, NP, SV, DS, JH, JS. All authors contributed to the article and approved the submitted version.

## Funding

JS, NP and JH are supported by the Wallace Fund of the New York Community Trust. JS is supported by a Damon Runyon Cancer Research Foundation Physician Scientist Training Award.

## Conflict of Interest

The authors declare that the research was conducted in the absence of any commercial or financial relationships that could be construed as a potential conflict of interest.
