# Supplementary material for: Changes in Urologic Operative Practice at the Beginning of the COVID-19 Pandemic in a Large, National Cohort
Source: Front Oncol. 2021 May 7;11:684787. doi: 10.3389/fonc.2021.684787 (PMC8138038; doi:10.3389/fonc.2021.684787)
Supplement: Supplementary file 1 [file DataSheet_1.docx]

**Supplementary list.** Radical cystectomy: "0TTB0ZZ", "0TTB4ZZ", "51999"; Nephroureterectomy: "0TT60ZZ", "0TT64ZZ", "0TT70ZZ", "0TT74ZZ", "50548", "50234", "50236"; Ureteroscopy: "0TC78ZZ", "0TC68ZZ", "0TC18ZZ","0TC48ZZ", "0TF78ZZ", "0TC08ZZ", "0TC38ZZ", "0TF68ZZ", "52356", "52352", "52353"; Stent only: "0T778DZ", "0T768DZ", "0T788DZ", "52332" (when not associated with ESWL or ureteroscopy); TURBT: "0TBB8ZX","0TBB8ZZ", "52235", "52234", "52240"; BPH procedures: "0VT08ZZ","0VB08ZZ", "0V508ZZ","0V507ZZ", "52601","52648","52649”; RALP: "0VT00ZZ", "0VT04ZZ", "55866" when not with “51999”; PCNL: "0TF33ZZ","0TC13ZZ", "0TC33ZZ", "0TF34ZZ", "0TC03ZZ", "0TC34ZZ", "0TF43ZZ", "0TC14ZZ", "0TC43ZZ", "0TF44ZZ", "0TC04ZZ", "0TC44ZZ", "50080", "50081"; Orchiectomy: "0VT90ZZ", "0VTB0ZZ", "0VTC0ZZ" when associated with oncologic/testis mass diagnoses: "N50.9", "C62.92", "C62.91", or "N50.89"; and "54530"; Partial nephrectomy: "0TB14ZZ", "0TB10ZZ", "0TB04ZZ", "0TB00ZZ", "50543", "50240"; Radical nephrectomy: "0TT00ZZ", "0TT04ZZ", "0TT10ZZ", "0TT14ZZ" when associated with oncologic/renal mass diagnoses: "C64.1", "C64.2", "C64.9", "N28.8", "N28.89", "N28.9" and not for donor purposes or ureteral tumor: "Z52.4", "C65.1", "C65.2"; Hydrocele: "55040", "55041", Sling: "57288", Penile prosthesis: "54405", "54400", Spermatocele: "54840", Circumcision: “0VTTXZZ”,

"54161"; Varicocelectomy: "55500", "55530", "55535".

**Supplementary table.**Multivariable models for absolute change in **(a)** overall procedure volume, **(b)** elective procedure volume, **(c)** urgent procedure volume, and **(d)** RALP and PNx volume.

Baseline volume was calculated for each hospital as average number of each procedure type, up to February 2020. Urban/rural and region were operationalized as defined by the US Census (and also by Premier). Teaching status was operationalized as defined by Premier; teaching hospitals have medical school affiliation and sponsor/participate in active residency programs. Proportion African-American patients was calculated as proportion of patients in total cohort self-reporting race as African-American for each hospital. Proportion Medicaid insurance patients was calculated similarly.

1. Overall

|  | Beta Estimate | Std. error | p-value |
| --- | --- | --- | --- |
| Intercept | 6.67409 | 2.07161 | 0.00139 |
| **Baseline average** | **0.20126** | **0.01227** | **< 0.001** |
| **Region- Midwest (vs. South)** | **-4.22940** | **1.88237** | **0.02526** |
| **Region- Northeast (vs. South)** | **-5.72310** | **2.27730** | **0.01241** |
| Region- West (vs. South) | -2.65747 | 2.68217 | 0.32246 |
| Academic (vs. non-academic) | -2.39185 | 1.73286 | 0.16837 |
| Urban (vs. rural) | -1.81643 | 1.82909 | 0.32135 |
| Percent African-American patients | 0.06666 | 0.08073 | 0.40953 |
| **Percent Medicaid insurance patients** | **-0.17016** | **0.08340** | **0.04207** |

(b) Elective only

|  | Beta Estimate | Std. error | p-value |
| --- | --- | --- | --- |
| Intercept | 0.604208 | 0.275163 | 0.02882 |
| **Baseline average** | **-0.401586** | **0.025742** | **<0.001** |
| **Region- Midwest (vs. South)** | **-0.688266** | **0.253688** | **0.00703** |
| **Region- Northeast (vs. South)** | **-0.636489** | **0.314657** | **0.04392** |
| Region- West (vs. South) | -0.547681 | 0.360424 | 0.12961 |
| Urban (vs.rural) | -0.472133 | 0.255192 | 0.06522 |
| Academic (vs. non-academic) | 0.202799 | 0.232510 | 0.38374 |
| Percent Medicaid insurance patients | 0.012784 | 0.012220 | 0.29628 |
| Percent African-American patients | -0.004341 | 0.011267 | 0.70025 |

(c) Urgent only

|  | Beta Estimate | Std. error | p-value |
| --- | --- | --- | --- |
| Intercept | 0.1378809 | 0.4249591 | 0.7458 |
| Baseline average | -0.0551154 | 0.0299264 | 0.0664 |
| Region- Midwest (vs. South) | -0.2086934 | 0.3798077 | 0.5830 |
| Region- Northeast (vs. South) | -0.0337425 | 0.4601377 | 0.9416 |
| Region- West (vs. South) | -0.3202825 | 0.5424484 | 0.5553 |
| Urban (vs.rural) | 0.3507465 | 0.3809785 | 0.3579 |
| Academic (vs. non-academic) | -0.4719488 | 0.3627415 | 0.1941 |
| Percent Medicaid insurance patients | -0.0089241 | 0.0176669 | 0.6138 |
| Percent African-American patients | 0.0004156 | 0.0181666 | 0.9818 |

(d) RALP and PNx

|  | Beta Estimate | Std. error | p-value |
| --- | --- | --- | --- |
| Intercept | 0.24615 | 0.41119 | 0.550 |
| **Baseline average** | **-0.17392** | **0.02222** | **<0.001** |
| Region- Midwest (vs. South) | -0.51477 | 0.35520 | 0.148 |
| Region- Northeast (vs. South) | -0.27708 | 0.42425 | 0.514 |
| Region- West (vs. South) | -0.44438 | 0.51058 | 0.385 |
| Urban (vs.rural) | 0.23802 | 0.39546 | 0.548 |
| Academic (vs. non-academic) | 0.52490 | 0.32463 | 0.107 |
| Percent Medicaid insurance patients | -0.01522 | 0.01661 | 0.360 |
| Percent African-American patients | -0.01733 | 0.01713 | 0.313 |
